# Supplementary material for: Pupil responses to social stimuli are associated with adaptive behaviors across the first 24 months of life
Source: J Neurodev Disord. 2025 Aug 1;17:44. doi: 10.1186/s11689-025-09628-2 (PMC12317559; doi:10.1186/s11689-025-09628-2)
Supplement: Supplementary file 1 — Supplementary Material 1. [file 11689_2025_9628_MOESM1_ESM.docx]

**Supplemental Summary Statistics**

**Table 1**

| **Summary of Variables Used in the First Stage Model** | | | | | | | | | | | | | | |
| --- | --- | --- | --- | --- | --- | --- | --- | --- | --- | --- | --- | --- | --- | --- |
| Variable | | Mean | | Std. dev. | | | Min | | Max | | |  |  |  |
| Pupil Size (mm) | | 4.00 | | 0.53 | | | 2.57 | | 5.53 | | |  |  |  |
| Fixation Time (ms) | | 1666.38 | | 2596.70 | | | 0 | | 13304.10 | | |  |  |  |
| Dynamic (Binary) | | 0.13 | | 0.34 | | | 0 | | 1 | | |  |  |  |
| Luminance | | 0.37 | | 0.15 | | | 0.00 | | 0.74 | | |  |  |  |
|  | | |  | | | | | | |  | | |  |  |
|  | Total | | | | Individual Average | | |  | | |  |  |  |  |
| Participants | 40 | | | | - |  | |  | | |  |  |  |  |
| Events | 18 | | | | 13.9 |  | |  | | |  |  |  |  |
| Observations (N) | 139,388 | | | | - |  | |  | | |  |  |  |  |

*Note: Any values less than 0.01(+/-) were set at 0.00, other than the means.

**Table 2**^12,15^

| **Summary of Variables Used in the Second Stage Model** | | | | | | | | | | | | |  |  |
| --- | --- | --- | --- | --- | --- | --- | --- | --- | --- | --- | --- | --- | --- | --- |
| Variable | | Mean | | | Std. dev. | | Min | | Max | |  |  |  |  |
| Pupil Size (mm) | | 4.00 | | | 0.47 | | 3.13 | | 5.09 | |  |  |  |  |
| VABS Composite Score | | 105.25 | | | 11.27 | | 83 | | 140 | |  |  |  |  |
| Social PRI (ms) | | -1.51x${10}^{-5}$ | | | 0.00 | | 0.00 | | 0.00 | |  |  |  |  |
| Non-Social PRI | | 1.16x${10}^{-5}$ | | | 0.00 | | 0.00 | | 0.00 | |  |  |  |  |
| Average Fixation Time (ms) | | 1525.34 | | | 795.85 | | 148.69 | | 3972.68 | |  |  |  |  |
| Age (months) | | 12.05 | | | 4.72 | | 6 | | 22 | |  |  |  |  |
|  |  | |  | | |  | |  | | | |  |  |  |
|  | Total | | |  | | |  | | |  | | |  |  |
| Observations (N) | 40 | | |  | | |  | | |  | | |  |  |

*Note: Any values less than 0.01(+/-) were set at 0.00, other than the means.

| **Table 3**  **First Stage Regression Results: Multi-Level Mixed Effects Model** | | | | | | | | | | | | | | | | | | | | | | | |  |  |
| --- | --- | --- | --- | --- | --- | --- | --- | --- | --- | --- | --- | --- | --- | --- | --- | --- | --- | --- | --- | --- | --- | --- | --- | --- | --- |
| Mixed Effects |  | |  | | | |  | | | |  | | | |  | | | |  | | | | |  |  |
|  | Coefficient | | | Std. err. | | z | | | | P>\|z\| | | | | 95% Confidence | | | | | | |  |  |  |  |  |
| Fixation Time (ms) | -1.51x${10}^{-4}$ | | | 0.00 | | -20.92 | | | | 0.00 | | | | 0.00 | | | 0.00 | | | | |  |  |  |  |
| Fixation Time ^2 (ms) | 9.74 x${10}^{-8}$ | | | 0.00 | | 29.20 | | | | 0.00 | | | | 0.00 | | | 0.00 | | | | |  |  |  |  |
| Fixation Time ^3 (ms) | -1.44 x${10}^{-11}$ | | | 0.00 | | -25.38 | | | | 0.00 | | | | 0.00 | | | 0.00 | | | | |  |  |  |  |
| Dynamic (Binary) | 5.35 x${10}^{-2}$ | | | 0.00 | | 29.17 | | | | 0.00 | | | | 0.05 | | | 0.06 | | | | |  |  |  |  |
| Dynamic * Fixation Time (ms) | 1.35 x${10}^{-4}$ | | | 0.00 | | 27.88 | | | | 0.00 | | | | 0.00 | | | 0.00 | | | | |  |  |  |  |
| Dynamic * Fixation Time^2 (ms) | -9.23 x${10}^{-8}$ | | | 0.00 | | -27.57 | | | | 0.00 | | | | 0.00 | | | 0.00 | | | | |  |  |  |  |
| Dynamic * Fixation Time^3 (ms) | 1.41 x${10}^{-11}$ | | | 0.00 | | 24.78 | | | | 0.00 | | | | 0.00 | | | 0.00 | | | | |  |  |  |  |
|  |  | | |  | | | |  | | | |  | | | |  | | | |  | | | | |  |
| Random Effects |  | |  | | | |  | | | |  | | | |  | | | |  | | | | |  |  |
|  | | Estimate | | | Std. err. | | | | 95% Confidence | | | | | | | | |  | | | | |  | | |
| *By Participant* | |  | | |  | | | |  | | | |  | | | | |  | | | | |  | | |
| Participant-Level Intercept | | 17.01 | | | 3.86 | | | | 10.91 | | | | 26.53 | | | | |  | | | | |  | | |
| *By Trial* | |  | | |  | | | |  | | | |  | | | | |  | | | | |  | | |
| Trial-Level Intercept | | 0.20 | | | 0.01 | | | | 0.18 | | | | 0.23 | | | | |  | | | | |  | | |
| Fixation Time (ms) | | 1.56 x${10}^{-8}$ | | | 0.00 | | | | 0.00 | | | | 0.00 | | | | |  | | | | |  | | |
| Luminance | | 1.46 | | | 0.10 | | | | 1.27 | | | | 1.67 | | | | |  | | | | |  | | |
| Covariance (Fixation Time, Luminance) | | -2.59 x${10}^{-6}$ | | | 0.00 | | | | 0.00 | | | | 0.00 | | | | |  | | | | |  | | |
| Covariance (Luminance, Constant) | | -0.45 | | | 0.03 | | | | -0.52 | | | | -0.38 | | | | |  | | | | |  | | |
| Covariance (Fixation Time, Constant) | | -5.26 x${10}^{-6}$ | | | 0.00 | | | | 0.00 | | | | 0.00 | | | | |  | | | | |  | | |
| Within-Trial Variance | | 0.02 | | | 0.00 | | | | 0.02 | | | | 0.02 | | | | |  | | | | |  | | |
|  |  | |  | | | |  | | | |  | | | |  | | | |  | | | | |  |  |
| Model Evaluation Metrics |  | |  | | | |  | | | |  | | | |  | | | |  | | | | |  |  |
| Adjusted $R^{2}$ | 0.92 | |  | | | |  | | | |  | | | |  | | | |  | | | | |  |  |
| RMSE | 0.15 | |  | | | |  | | | |  | | | |  | | | |  | | | | |  |  |

*Note: Any values less than 0.01(+/-) were set at 0.00, other than the coefficients.

**Table 4**

| **Second Stage Regression Results: Predict VABS Composite Scores per Participant** | | | | | | | |
| --- | --- | --- | --- | --- | --- | --- | --- |
|  | Coefficient | Std. err. | t | P>t | 95% Confidence | |  |
| Social PRI (Fixation Time (ms)) | 89978.08 | 41837.11 | 2.15 | 0.038 | 5044.23 | 174911.90 |  |
| Non-Social PRI (Fixation Time (ms)) | 24954.96 | 24499.46 | 1.02 | 0.315 | -24781.60 | 74691.51 |  |
| Age | -0.89 | 0.36 | -2.52 | 0.017 | -1.61 | -0.17 |  |
| Average Fixation Time (ms) | 1.20 x${10}^{-5}$ | 0.00 | 0.01 | 0.996 | 0.00 | 0.00 |  |
| Constant | 117.10 | 5.31 | 22.06 | 0.000 | 106.33 | 127.88 |  |
|  |  |  |  |  |  |  |  |
| Model Evaluation Metrics |  |  |  |  |  |  |  |
| Adjusted R2 | 0.17 |  |  |  |  |  |  |
| RMSE | 10.26 |  |  |  |  |  |  |

*Note: Any values less than 0.01(+/-) were set at 0.00, other than the coefficients.
